# Incidence and progression of myopia and associated factors in urban school children in Delhi: The North India Myopia Study (NIM Study)

**DOI:** 10.1371/journal.pone.0189774

**Published:** 2017-12-18

**Authors:** Rohit Saxena, Praveen Vashist, Radhika Tandon, Ravindra M. Pandey, Amit Bhardawaj, Vivek Gupta, Vimala Menon

**Affiliations:** 1 Department of Ophthalmology, Dr Rajendra Prasad Center for Ophthalmic Sciences, All India Institute of Medical Sciences, New Delhi, India; 2 Department of Community Ophthalmology, Dr Rajendra Prasad Center for Ophthalmic Sciences, All India Institute of Medical Sciences, New Delhi, India; 3 Department of Biostatistics, All India Institute of Medical Sciences, New Delhi, India; Soochow University Medical College, CHINA

## Abstract

**Aim:**

To evaluate the incidence and progression of myopia and factors associated with progression of myopia in school going children in Delhi.

**Methods:**

Prospective longitudinal study of 10,000 school children aged 5 to 15 years screened after an interval of 1 year to identify new myopes (Spherical Equivalent≤ -0.5D) and progression of myopia in previously diagnosed myopic children. Association between risk factors and progression was analyzed using adjusted odds ratio.

**Results:**

Of the 9,616 children re-screened (97.3% coverage), annual incidence of myopia was 3.4%with mean dioptric change of -1.09 ± 0.55. There was a significant higher incidence of myopia in younger children compared to older children (P = 0.012) and among girls compared to boys (P = 0.002). Progression was observed in 49.2%children with mean dioptric change of -0.27 ± 0.42 diopters. The demographic and behavioral risk factors were analyzed for children with progression (n = 629) and adjusted odds ratio values were estimated. Hours of reading-writing/week (p<0.001), use of computers/ video games (P<0.001) and watching television (P = 0.048) were significant risk factors for progression of myopia. Outdoor activities / time spent outdoors> 2 hours in a day were protective with an inverse association with progression of myopia (P< 0.001).

**Conclusion:**

Myopia is an important health issue in India and is associated with long hours of reading and screen time with use of computers and video games. An annual eye vision screening should be conducted, and outdoor activities be promoted to prevent the increase of myopia among school children.

## Introduction

Myopia is one of the most common ocular disorders seen in children and young adults and is a cause of concern world-over [1–4]. While the prevalence of myopia has been reported to be very high in East Asia [5–16], it is as yet not considered a cause for concern in India. A previous study by the authors had reported a prevalence of only 13.1% among school children in India [17] which is very low compared to that reported from other regions of Asia. However, this is higher than most previous reports from India [18,19] and may reflect a progressive increase in the prevalence of myopia in India. Moreover, the conditions that have been associated with an increased risk of developing myopia i.e. competitive educational environment, long study hours and reduced outdoor activity [4,20–22] are commonly observed in Indian cities. While there are studies on the incidence and progression of myopia from East Asia [23–29] and population with European ancestry [30–32], there is no longitudinal study from India. As studies on the incidence of myopia are essential to determine differences in risk between populations, the aim of the current study was to assess the incidence and progression of myopia in school going children in Delhi and to compare them with published data from other sources. This is a follow up of a previous study which had established the prevalence of myopia in the same cohort [17].

## Methods

The North India Myopia Study (NIM Study) was a school based cohort of children from 20 schools in Delhi who were examined to determine the prevalence of refractive errors [17].

### Study population

The same cohort of children were re-examined after a period of one year to evaluate the incidence and progression of myopia.

The study was approved by the Institutional Ethics Committee of our hospital and followed the tenets of the Declaration of Helsinki for biomedical research. Permission for conducting the study in the selected schools was taken from the District Education Authority. A form in English and the local vernacular language which is Hindi was sent to all the parents to sign for providing the informed consent for the procedure. If the consent form was not returned or the parents had any doubts regarding the procedure, they were contacted by telephone and all concerns regarding the study were addressed. If the parents still did not return the signed informed consent form, the child was not enrolled in the study.

The previously compiled list of all the enrolled children in the school in classes 1 to 10 was used with the Unique Identification number (U.I.D. Number) that was given in the first round. As most of the children were in the subsequent higher class, the rolls were compared with the roll list of the subsequent class and the U.I.D. numbers were matched to ensure that the details of the same child are recorded in the evaluation sheet. The help of the respective class teacher was taken to ensure correct identification of each child. The list of children that had left school over the previous 1 year was matched with the list of enrolled children and they were removed from the list for examination.

Wherever possible the teacher who had participated in the study initially in the first year was asked to assist in the follow up re-examination of the children. Prior participation of teachers in the original study had made them familiar with the examination process and the study protocol which helped to minimize time spent in training of the teachers and explaining the process of examination. The most important function of the teachers was to ensure correct identification of the child with the Unique Identification number given to the child in the first round.

### Visual assessment

The vision of the child was documented by the primary eye care worker who was unaware of the findings of the first round and of the refractive status of the child unless he / she wore glasses. The visual acuity of each was assessed while the fellow eye was closed with the palm of the hand. The vision level of the child measured by the primary eye care worker was recorded as the line with the smallest letters read accurately by the child.

All those children who had normal unaided vision (6/9.5 or better) were sent back to the class. Children with sub-normal visual acuity i.e. those unable to read the 6/9.5 line on the ETDRS chart and those children having previous myopic glasses were further examined by an optometrist for confirmation of vision and refraction if required. Refraction was done in 2 stages by a single trained optometrist, first under cycloplegia using eye drops 2% homatropine which was instilled in the inferior conjunctival cul-de-sac twice at an interval of ten minutes. If the pupillary light reflex was still present after 20 minutes, a third drop was administered. Cycloplegia was considered complete if pupil was dilated to 6 mm or more and there was no pupillary light reflex. Retinoscopy was done using a streak retinoscope and a hand held autorefractometer (Retinomax K-Plus; Nikon Corp., Tokyo, Japan). The autorefractometer was calibrated at the beginning of each working day and a single reading was taken for each eye. Based on the findings of the refraction under dilatation, subjective refraction was done at a following visit after a week. The final prescription was based on the subjective refraction. All children prescribed spectacles were provided the same at concessional rate.

The current examination findings were compared with the findings of the first round. All the children diagnosed as myopic in the first round formed the denominator for evaluation of progression of myopia. All those children who had normal unaided presenting vision in the first round but failed to read the 6/9.5 line on the ETDRS chart were the new cases of refractive error and were evaluated for identifying cases with myopic refractive error to determine the incidence of myopia.

#### Working definition

Spherical equivalent (SE) values were derived by adding the sum of the sphere power with half of the cylinder power. Incidence was defined as the number of children diagnosed with myopia (SE ≤ -0.5D) on re-screening after a period of 1 year among children with normal vision in the baseline examination. Progression was defined as any increase in the spherical equivalent of the refractive error of the child over a period of 1 year.

All the children wearing glasses or those who were identified to be myopic in round one were re-evaluated in round two. Of these, children who were unable to read the 6/9.5 line on the ETDRS chart were refracted by the optometrist and if there was any increase in the myopic power, were included in the list of cases with progression.

#### Questionnaire

For all children diagnosed with progression of myopia a structured questionnaire regarding risk factors was filled. The questions were asked in Hindi which is understood by all the children and parents and the answers were recorded in English. The questionnaire was filled by asking the details from the child and by telephonic interview of one or both parents. For collecting data on the hours spent in the various activities the actual total time spent for the activity in school and at home was recorded. The question on hours spent outdoors was aimed to capture the entire time spent outdoors and not just for sports and recreational activities.

#### Data analysis

The statistical analysis was carried out using Stata 14.0 (College Station, USA). The data was presented as number (%) or mean ± SD as appropriate. The incidence of myopia was presented as percentage (95% Confidence Interval-CI). We calculated the cumulative incidence rate (number of incident cases divided by the number at risk at baseline, i.e. those who are not myopic at baseline). Progression of myopia was reported as percentage showing progression and the mean progression observed. The risk factors for progression were divided into demographic (non-modifiable) and behavioral (modifiable) risk factors. Logistic regression analysis was carried out to find the adjusted odd ratio for various risk factors for progression of myopia. The factors adjusted for were: age; gender, type of school, socioeconomic status, parental use of distance spectacles, hours of reading/writing at school and home, watching television, using computers / video games and outdoor activity. The results were reported as Adjusted Odds Ratio (95% CI). A P-value < 0.05 was considered statistically significant.

## Results

All the children who were examined in round one (n = 9,884) were enrolled for the second round of examination and 9,616 children (coverage of 97.3%) could be screened. The rest were absent on all the days that the screening was being done in that particular school and also on two subsequent re-visits within a 2-week period.

**Fig 1:**
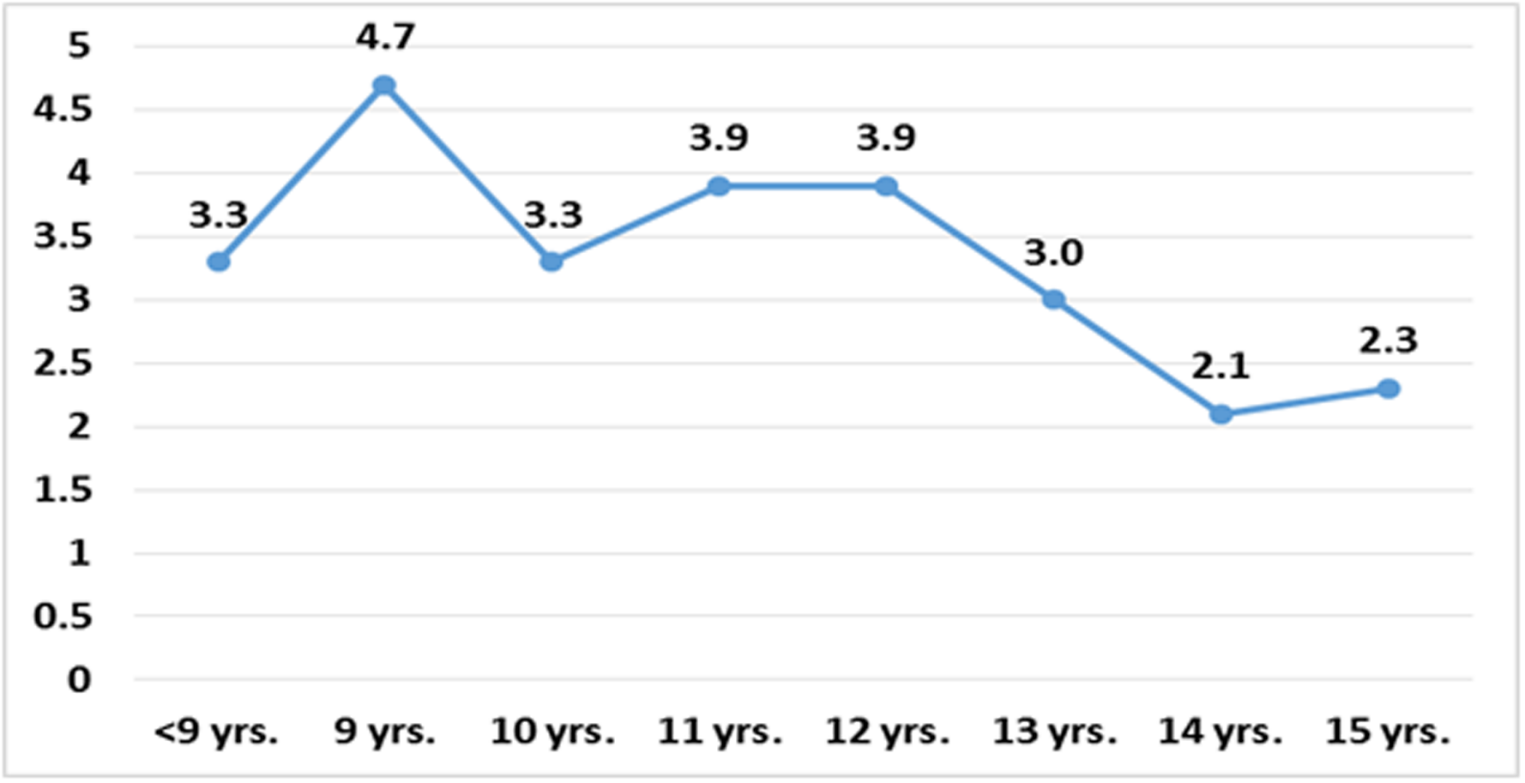
Age specific incidence of myopia (n = 8200).

There were 8200 children with normal unaided visual acuity in the first round and after an interval of one year, 275 children (3.4% SE 0.2, 95% CI 3.0–3.8) developed myopia of spherical equivalent ≤ -0.5 D. The mean dioptric power observed in the children who developed myopia was -1.09 ± 0.55 (SE 0.033, 95% CI [(-1.16)—(-1.03)]). There was a significant higher incidence of myopia in younger children compared to older children (P = 0.012) and among girls compared to boys (P = 0.002). No association of type of school (private schools vs. government schools) was observed on the incidence of myopia (Table 1). Age specific incidence of myopia is shown in Fig 1 and Table 2. The mean age of onset of formal education was 6.3±1.3 years.

**Table 1:** Distribution of age, gender and type of school with incidence and progression of myopia.

	**Incidence cases n = 275(%)**	**Total n = 8200**	**95% CI**	**P**
**Age in year**				
5–10	103 (3.7)	2768	3.0, 4.4	**0.012**
11–13	136 (3.6)	3783	3.0, 4.1	
14–15	36 (2.2)	1649	1.4, 2.8	
**Gender**				
Boy	162(2.9)	5528	2.4, 3.3	**0.002**
Girl	113(4.2)	2672	3.4, 4.9	
**Type of School**				
Government School	109(3.0)	4544	2.4, 3.5	0.093
Private School	166(3.6)	3656	3.1, 4.1	
	**Progression (%) n = 629**	**Total n = 1279**	**95% CI**	**P**
**Age in year**				
5–10	138(52.3)	264	46.2–58.3	0.470
11–13	337(47.9)	704	44.2–51.6	
14–15	154(49.5)	311	43.9–55.1	
**Gender**				
Boy	390(48.2)	810	44.6–51.6	0.332
Girl	239(51.0)	469	46.4–55.5	
**Type of School**				
Government School	161(50.0)	322	44.5–55.5	0.733
Private School	468(48.9)	957	45.7–52.1	

**Table 2:** Incidence of myopia according to age.

Age in years	N	No. of new cases of myopia (%)
<9	860	28 (3.3)
9	859	40 (4.7)
10	1049	35 (3.3)
11	1253	49 (3.9)
12	1310	51 (3.9)
13	1220	36 (3.0)
14	984	21 (2.2)
15	665	15 (2.3)
**Total**	**8200**	**275 (3.4)**

Of the 1297 children diagnosed with myopia in the first round, 18 children were absent in second round. Of the1279 children examined, 629 (49.2%)children showing increase in myopia during one-year period. The mean rate of myopic progression was (-0.27± 0.42 D; SE 0.01) 95% CI[(-0.24) to (-0.42)].

**Fig 2:**
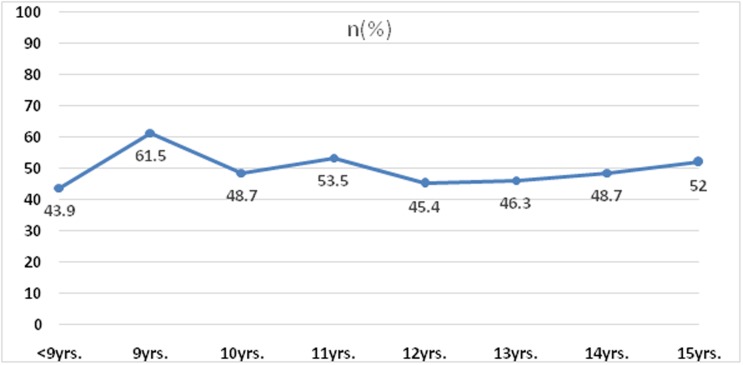
Age specific progression of myopia (n = 1279).

The distribution of the number of cases with different amount of progression of myopia is given in Table 3. The number of cases showing myopic progression of ≥ -0.5D were360 (28.2%). Age specific progression of myopia is shown in Fig 2 and Table 4. There was no significant association of progression of myopia with the severity of myopia (P = 0.6), age (P = 0.470), gender (P = 0.332) or type of school (P = 0.733). Table 1.

**Table 3:** Frequency of cases with quantum of myopia progression after 1 year.

Change in spherical equivalent (D)	No. of children with progression (%)
-1.50	33 (2.6)
-1.25	24(1.9)
-1.00	37(2.9)
-0.75	61(4.8)
-0.50	205 (16.0)
-0.25	269(21.0)
0	650 (50.8)
**Total**	**1,279 (100.0)**

**Table 4:** Progression of myopia according to age.

Age in years	n	No. of children with progression (%)
<9	49	21(42.9)
9	96	59(61.5)
10	119	58(48.7)
11	185	99(53.5)
12	238	108(45.4)
13	281	130(46.3)
14	234	114(48.7)
15	77	40(52.0)
**Total**	**1279**	**629(49.2)**

The mean duration of hours per week spent in the various behavioral risk factors is given in Table 5. Age distribution of mean hours spent in reading / writing and outdoor activity is given in Table 6. A multifactorial regression analysis was done to identify the demographic and behavioral risk factors for progression. Table 7.

**Table 5:** The mean and range of the hours spent per week in various behavioral risk factors among children with progression.

N = 1279	Mean ± SD (hours /week)	Range
**Outdoor activity**	13.95 ± 1.9	8–24
**Read / writing**	41.63 ± 3.87	32–54
**TV**	18.15 ± 6.15	2–35
**Video games**	5.17 ± 3.82	0–22

**Table 6:** Age distribution of mean hours spent in reading / writing and outdoor activity.

Age	N	Outdoor activity (mean ± SD)	Reading/ writing (mean ± SD)
7	35	15.1 ± 3.2	39.0 ± 3.5
8	151	14.7± 3.1	38.8 ± 3.2
9	235	13.9 ± 2.5	40.1 ± 4.0
10	306	14.5 ± 3.0	41.0 ± 4.1
11	421	13.7 ± 2.9	41.8 ± 4.1
12	477	13.5 ± 3.2	41.8 ± 4.0
13	519	13.6± 3.5	42.6 ± 4.1
14	412	13.6 ± 3.3	42.3 ± 4.2
15	186	14.1 ± 3.4	41.1 ± 4.1

**Table 7:** Association of progression of myopia adjusted for different demographic and behavioral factors.

Progression Denominator (n = 1279)	Progression (%) n = 629	No Progression (%) n = 650	Adjusted OR (95% CI)	P- Value
**Age in Years**				
5–10	138(21.9)	126(19.4)	1	
11–13	337(53.6)	367(56.5)	1.2 (0.84–1.57)	0.356
14–15	154(24.5)	157(24.2)	1.1 (0.79–1.64)	0.482
**Gender**				
Boy	390(62.0)	420(64.6)	1	
Girl	239(38.0)	230(35.4)	1.1 (0.84–1.40)	0.503
**Type of School**				
Government School	161(25.6)	161(24.8)	1	
Private School	468(74.4)	489(75.2)	1.1 (0.77–1.45)	0.690
**Socioeconomic status**				
Lower to Upper Lower	67(10.9)	72(11.3)	1	
Lower Middle	185(30.2)	226(35.6)	0.9 (0.60–1.42)	0.739
Upper Middle to Upper	361(58.9)	337(53.1)	1.0 (0.62–1.48)	0.861
**History of parental use of distance spectacles**				
Not wearing spectacles	538(85.5)	561(86.3)	1	
Wearing spectacles	91(14.5)	89(13.7)	1.2 (0.84–1.67)	0.316
**No. of hours per week of reading/writing at school and home**				
28–35	30(4.8)	70(10.9)	1	
36–42	373(59.3)	413(64.0)	1.62(0.98–2.67)	0.058
>42	226(35.9)	162(25.1)	2.10(1.24–3.56)	**0.006**
**No. of hours per week of watching television**				
≤14	26(4.1)	54(8.4)	1	
15–21	228(36.3)	345(53.5)	1.0 (0.57–1.71)	0.997
>22	375(59.6)	246(38.1)	1.6 (0.92–2.83)	0.083
**No. of hours per week of using computers and video games**				
0–4	134(21.3)	294(45.6)	1	
4–7	278(44.2)	256(39.7)	1.89(1.42–2.49)	**<0.001**
>7	217(34.5)	95(14.7)	3.53(2.51–4.95)	**<0.001**
**No. of hours per week of outdoor activity**				
≤14	580(92.2)	515(79.8)	1	
> 14	49(7.8)	130(20.2)	0.54(0.37–0.79)	**0.002**

The following factors were adjusted for: age; gender, type of school, socioeconomic status, parental use of distance spectacles, hours of reading/writing at school and home, watching television, using computers / video games and outdoor activity. The results showed a significant positive association of progression of myopia with greater number of hours of reading/writing per week, and use of computers/ video games. No association was observed between progression and age, gender, type of school, parental history of use of glasses socio-economic status and hours of watching television. Outdoor activity/ time spent outdoors per week at school and at home were protective as an inverse association of progression was observed with outdoor activities/playing > 2 hours in a day (P< 0.001). We evaluated the correlation between TV and outdoor activity. A weak inverse correlation was observed (r^2^ = -0.25; P = 0.01) showing that those children who were watching TV for longer hours were less likely to play outdoors. No such correlation was observed between near work and outdoor activity.

## Discussion

This was a prospective longitudinal study to re-screen school going children for incidence and progression of myopia with coverage of 97.3%after a period of 1 year. To the best of our knowledge this is the first longitudinal cohort study to report incidence and progression of myopia in the Indian population. According to the report of the Directorate of Economics and Statistics Government of National Capital Territory of Delhi 2016, the Gross Enrolment Ratio in primary and secondary schools in Delhi are 114 and 103 respectively. Therefore, it is expected that most of the children of 5–15 years in the districts surveyed would be attending school. As we had coverage of 97.3% we expect that our sample should be representative of the school going population in Delhi [33].

The results show that the annual cumulative incidence of myopia was 3.4% which is much lower than those reported in East Asia [23–29]and appears to be comparable to Caucasian children [30–32]. Incidence was more in younger age group and among girls. There was no effect of the type of school on the incidence of myopia.

The results showed a statistically significant higher incidence with younger age. The incidence increased by8-9 years of age and remained high till around 12 years after which a decrease was observed. While studies have shown a progressive increase of incident myopia with age [11, 34], one possible reason for this finding in our study could be the early introduction of reading and writing in urban schools in India. The mean age of onset of formal education was 6.3 years for children in our study and many children reported receiving non-formal pre-school education. With higher incidence and prevalence as reported in this study, girls appear to be at greater risk than boys. Similar findings have been reported by previous studies [11, 31, 34]. We have already reported that girls in India tend to read and write more and spend a greater amount of time indoors [17]. This increase in near-work possibly predisposes them to development of myopia.

The results showed that nearly 50% of the children had a progression in myopia with a mean increase of -0.27 D of myopia over a period of 1 year. Though these rates are much lower than those reported from other Asian regions, it is comparable to those from Caucasian studies. A reason could be that the Indian population may be genetically closer to the Caucasian population. Alternatively, even in urban areas, India may not have such a highly competitive educational environment as is evident in parts of East Asia with high prevalence of myopia.

According to the report of the Directorate of Economics and Statistics Government of National Capital Territory of Delhi 2016, the Gross Enrolment Ratio is lower in secondary schools compared to primary schools. This shows that some children drop out in higher classes as they are unable to cope with the increasing academic pressures and there may be selective retention of high performing students. Moreover, the mean hours spent in reading/writing was similar across all ages therefore we do not see the drop of progression of myopia with age as is seen in other populations.

The results showed that>6hours of reading/writing per day and >4 hours of playing video games per week showed a significant positive association with progression of myopia. Time spent outdoors per week at school and at home was protective as an inverse association of progression was observed with time spent outdoor /outdoor activity> 2 hours in a day. There was no correlation of near activity with outdoor activity showing that they are independent risk factors for development of myopia and one is not due to absence of the other.

While both prevalence and incidence of myopia appear to be inversely related to time spent outdoor, progression studies have not shown a similar association [35,36]. French et al have suggested that failure to be able to have a clear association between time outdoors and progression of myopia may be a statistical issue as most myopic children tend to engage in greater hours of near activities and spend less hours outdoors. This reduces the variability in their behavior needed to establish association [37]. In our study we observed that >2 hours/day of outdoor activity can be protective for progression of myopia.

The strength of the current study is its large sample size and the high coverage. However, as the data has been collected from a single city it may not be reflective of the entire country especially due to the known difference in myopia between urban and rural regions. Cycloplegic refractions were done on children only if their VA was < 6/9.5. While this has been reported to be the best cut-off for predicting myopia [38], but there will be some children who were actually myopic at baseline and follow-up but may have been missed thus underestimating the true incidence. There would also be some children who despite small increase in the myopic power were able to read the 6/9.5 line and would have been missed at the time of screening. This is an inherent limitation of using a visual acuity as a screening tool to identify increase in refractive error and may have resulted in a small underestimation of progression. Also, data for the risk factors was collected using a questionnaire based system which has inherent limitations. Though a multi-factorial analysis was performed to evaluate risk factors for progression of myopia, there may be an overlap of the effect of commonly associated risk factors.

Our study shows that myopia is an important health issue in India with high incidence and progression. In India, the School Vision Screening Programme, part of the National Programme for Control of Blindness, is an important strategy for controlling visual impairment due to refractive error. However, no guidelines exist for the frequency of screening which is usually done after 3–4 years or as per the availability of resources which vary in different parts of the country. Based on these rates we recommend that school screening should be conducted annually and that the prevalence of myopia in this population is likely to show a marked increase which can have major implications to the financial cost of correcting these refractive errors. It is evident that there is a progressive change in the lifestyle of urban children in India with increased tendency to remain indoors and engage in technological devices for entertainment. It is therefore imperative that some policy changes be implemented in the school curriculum so that increase in outdoor activity may be incorporated in the daily timetable. This may not only help to reduce the magnitude of myopia but also may help in the general health and well-being of the growing child.
